# Unexpected *Mycoplasma hominis* infection in two renal transplant recipients traced back to the same donor by whole-genome sequencing

**DOI:** 10.1007/s10096-020-04116-y

**Published:** 2020-12-23

**Authors:** V. Hinić, H. M. B. Seth-Smith, S. Damm, P. Amico, N. Khanna, A. Egli, V. Bättig

**Affiliations:** 1 Division of Clinical Bacteriology and Mycology, University Hospital Basel, Basel, Switzerland; 2 Applied Microbiology Research, Department of Biomedicine, University of Basel, Basel, Switzerland; 3 Clinic for Transplantation Immunology and Nephrology, University Hospital Basel, Basel, Switzerland; 4 Division of Infectious Diseases and Hospital Epidemiology, University Hospital Basel, Basel, Switzerland

**Keywords:** *Mycoplasma hominis*, Renal transplant, Typing, Whole-genome sequencing (WGS)

## Abstract

*Mycoplasma hominis* is a common colonizer of the lower genitourinary tract. Although its clinical relevance for causing urogenital infections in immunocompetent individuals is controversial, this bacterium has been involved in severe invasive infections in allograft recipients. In this report, we describe two cases of *M. hominis* infection in two young renal transplant recipients within the first month post-transplant. Although at first no epidemiological link between the two cases had been suspected, whole-genome sequencing (WGS) analysis showed that both isolates were identical, highly suggestive of an origin with the common organ donor.

**Supplementary Information:**

The online version contains supplementary material available at 10.1007/s10096-020-04116-y.

## Introduction

*Mycoplasma hominis* belong to the class of *Mollicutes*, are the smallest self-replicating prokaryotes, and are characterized by the absence of a cell wall. Since *M. hominis* is frequently detected in the lower urogenital tract in sexually active healthy men and women, the understanding of their etiologic role as a pathogen is somewhat controversial [1]. Although infections in immunocompetent patients have been reported [2, 3], most publications implicate *M. hominis* in invasive disease in immunosuppressed persons [4–8].

In contrast to other *Mycoplasma* spp., *M. hominis* can grow on conventional bacteriological media. However, the growth occurs often only after prolonged incubation, and, due to their morphology, the fine colonies can be easily overlooked especially in the presence of concomitant contaminating flora.

In this report, we describe two cases of extragenital infection with *M. hominis* in two renal transplant recipients, discovered accidentally in the bacterial culture. The epidemiological link between the two cases has been established by whole-genome sequence (WGS) analysis.

## Case 1

**Fig. 1:**
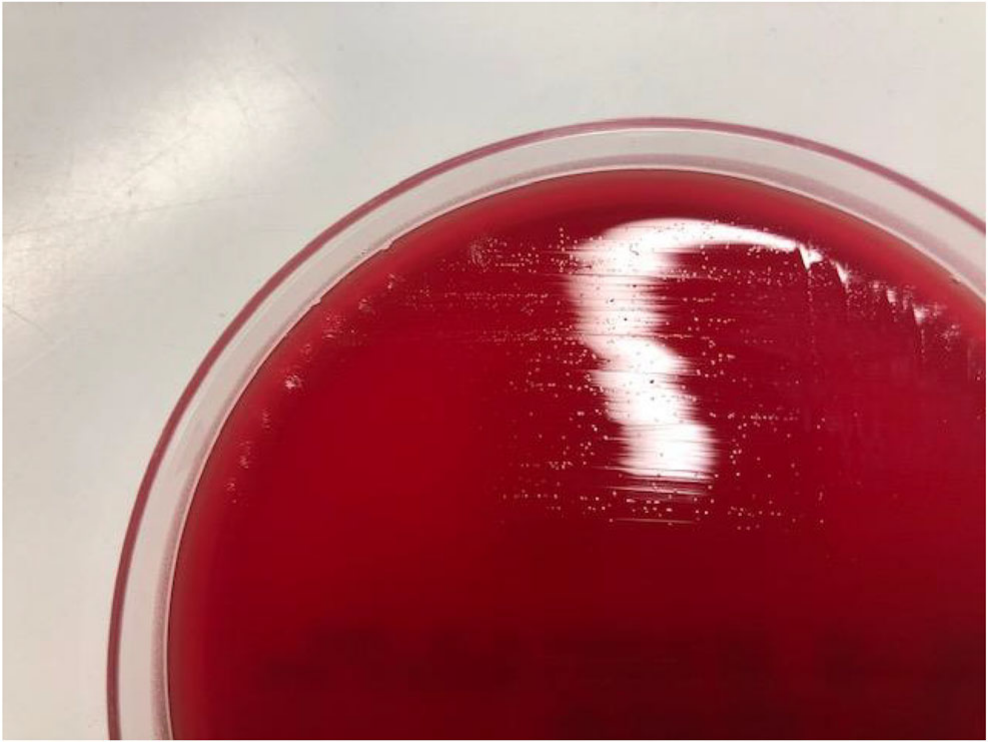
Growth of *M. hominis*. Picture was taken after 6 days of incubation on *Brucella* agar under anaerobic conditions

A 19-year-old patient with history of diethylene glycol poisoning–induced end-stage renal disease underwent kidney transplantation from a deceased donor. Three days later, a relaparotomy was performed because of postoperative bleeding with consecutive retroperitoneal hematoma. Due to persistent fever and elevated inflammation markers, antibiotic treatment with piperacillin–tazobactam was started with inadequate response. A computed tomography (CT) scan of abdomen and pelvis 2 weeks after the transplantation revealed progredient subcutaneous epifascial fluid collection along the surgical suture, compatible with postoperative residual hematoseroma with possible secondary infection. The collection was drained and an aspirate sent to the microbiology laboratory for bacterial culture. Direct Gram stain revealed copious leukocytes and erythrocytes, but no bacteria. On the sixth day of incubation, small translucent colonies on *Brucella* agar with 5% horse blood (BD) incubated under anaerobic atmosphere were observed (Fig. 1, isolate 612321-19). 16S rRNA gene sequencing identified the bacteria as *M. hominis*. Upon receipt of microbiology result, the antimicrobial therapy was changed to doxycycline 100 mg twice daily per os for 7 days. All blood cultures and urine samples remained without growth.

**Fig. 2:**
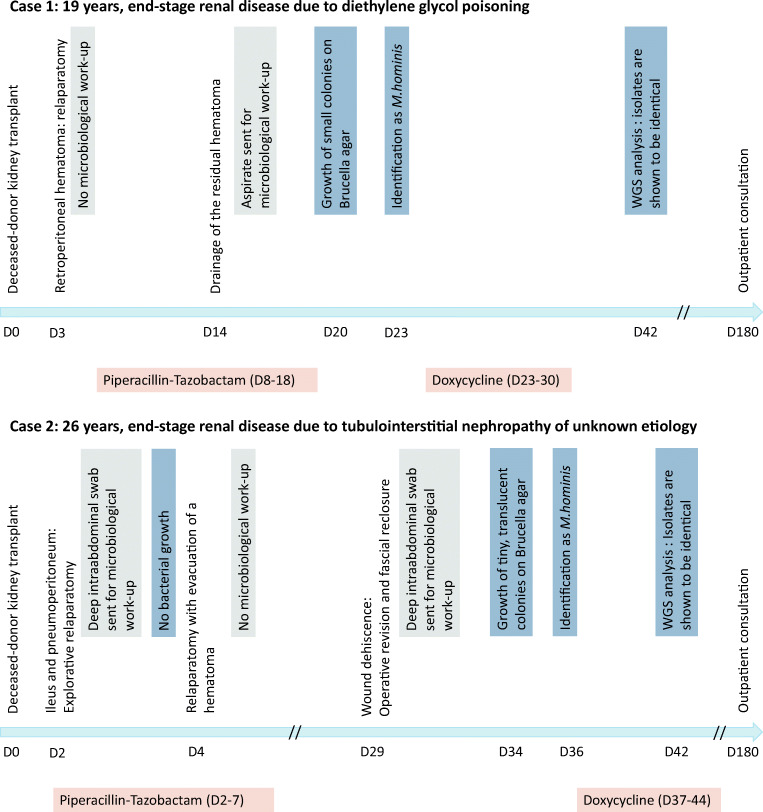
Timeline for case 1 and case 2

Under adequate treatment, inflammation parameters completely normalized and the patient was discharged home. In the last outpatient consultation 6 months after the transplantation, the patient was well with no sign of infection and the kidney function was stable at an eGFR of 71 ml/min/1.73 m^2^ calculated by CKD-EPI (Chronic Kidney Diesaese- Epidemiology Collaboration) without proteinuria. See timeline in Fig. 2.

## Case 2

A 26-year-old man received a kidney transplantation from a deceased donor due to tubulointerstitial nephropathy of unknown etiology. On day two post-transplantation, the patient developed clinical signs of ileus with acute abdominal pain and emesis. CT scan indicated a pneumoperitoneum, raising suspicion about intestine perforation. On the same day, explorative laparotomy was performed, whereby organ perforation could be excluded, so that the pneumoperitoneum seen was associated with the peritoneal dialysis. A deep intraabdominal swab from the laparotomy site and blood cultures were collected and showed no bacterial growth. Due to diffuse hemorrhage originating from the surgical wound, a further laparotomy with evacuation of a hematoma in the abdominal wall was necessary 2 days later. At day 26 after transplantation, the patient was discharged home. In the first outpatient consultation a few days later, a wound dehiscence was seen with consecutive operative revision once again and fascial reclosure. Intraoperatively, two deep intraabdominal swabs were obtained. Direct Gram stain was performed but showed no presence of bacteria. On the fifth day of incubation, tiny, translucent colonies were observed on *Brucella* agar with 5% horse blood incubated under anaerobic atmosphere (isolate 613095-19). Again, 16S rRNA gene sequencing identified the isolate as *M. hominis*. Accordingly, doxycycline 100 mg twice daily was initiated for 7 days with excellent clinical response. In the last outpatient consultation 6 months after the transplantation, the patient was well with no sign of infection and the kidney function was stable at an eGFR of 61 ml/min/1.73 m^2^ calculated by CKD-EPI without proteinuria. See timeline in Fig. 2.

**Fig. 3:**
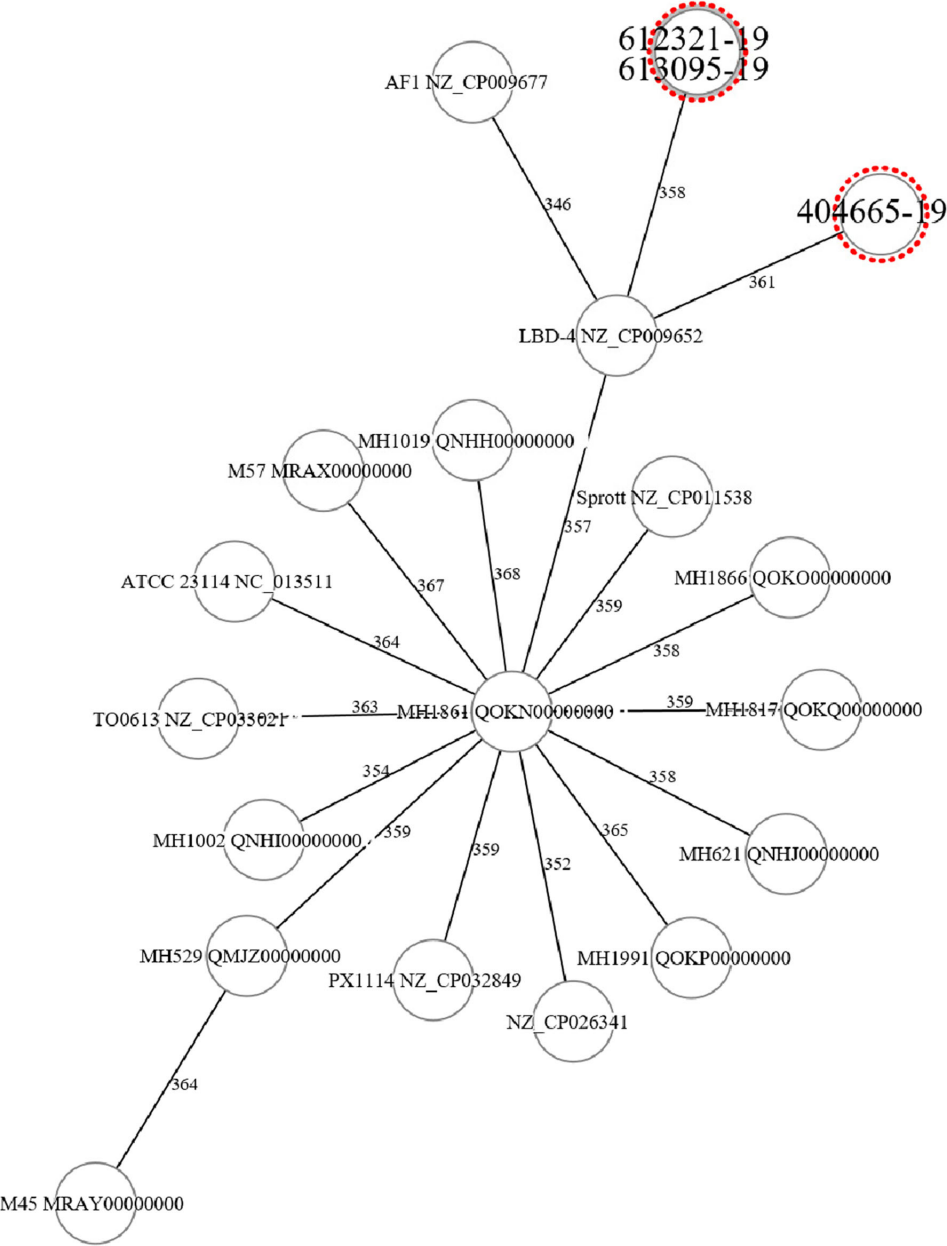
Minimum spanning tree based on cgMLST analysis of *M. hominis* genomes. Figure was generated in Ridom Seqsphere+ based on an ad hoc scheme using 413 targets. Distances between genomes show the number of allele differences. The two case isolates are 612321-19 and 613095-19, and are seen to be identical, and the control isolate is 404665-19. Other genomes from databases are shown for context

## Microbiological and whole-genome sequencing analysis

Intraoperative specimens from both patients were processed routinely at the microbiology laboratory including Gram stain and culture on Columbia agar with 5% sheep blood (BD) and Chocolate agar PolyViteX (bioMérieux) incubated aerobically for 2 days, fluid thioglycolate medium (BD) incubated aerobically for 6 days, and *Brucella* agar with 5% horse blood (BD) incubated for 6 days under anaerobic conditions. Aerobic and anaerobic blood cultures obtained from both patients were incubated for 5 days in a BacT/ALERT® VIRTUO® detection system (bioMérieux). 16S rRNA gene sequencing was performed as described previously [9]. The two case strains were isolated within 16 days of each other. DNA from both isolates, and an unrelated *M. hominis* isolate from the same time period as a control (404665-19), was extracted by Qiagen EZ1. WGS was performed on an Illumina NextSeq500 2x150bp after Nexteraflex library preparation, providing mean coverage over 69 ×. An ad hoc core genome MLST (cgMLST) scheme based on the ATCC 23114 genome (FP236530) and 16 further query genomes (Table S1) was calculated in Ridom Seqsphere+ v 6.0.2 (https://www.ridom.de/seqsphere/u/Tutorial_for_Ad_hoc_cgMLST_Schema.html) producing a scheme with 413 targets (Table S2). cgMLST analysis (Fig. 3) and further complete genome SNP analysis in CLC Genomics Workbench v20.0.2 using the genome of isolate 612321-20 as the reference (data not shown) show that the two case isolates are identical, in contrast to the control isolate and further database isolates. All NGS data has been deposited under project PRJEB40931.

## Discussion

*Mycoplasma hominis* has been reported as a cause of serious infections in allograft recipient patients, mostly kidney recipients [4, 6–8, 10]. The incidence of infections may even be underrecognized, due to difficulties in detection of this bacterial group with conventional microbiological diagnostic procedures. Culturing *Mycoplasma* spp. is laborious, usually requiring special growth media, and, depending on species, can take up to several weeks [11, 12]. *M. hominis* is the only pathogenic *Mycoplasma* of human origin that can grow on routinely used bacteriologic blood-based media in both aerobic and anaerobic conditions and for which growth is usually observed in 2 to 6 days from primary specimens. Still, they are often difficult to find in the routine culture due to the following reasons: (a) the organism is never recognized in the direct Gram stain, since there is no cell wall; (b) the colonies are tiny and translucent, and can be mistaken for material (fat droplets from the tissue); and (c) colony growth is visible not before 48 h of incubation. In both cases described here, growth was observed only after 5 or 6 days of incubation. This was only possible as our laboratory routinely incubates anaerobic plates from intraoperative samples from primary sterile sites for longer period of 6 days (in contrast, aerobic cultures are incubated for 2 days); in most laboratories, both aerobic and anaerobic cultures are incubated for only 2 days. Additionally, *Mycoplasma* colonies can be easily overlooked if overgrown by other microbiota present in the sample. Therefore, detection of *M. hominis* should not normally be relied upon in routine bacteriological culture.

Blood cultures collected from both patients were negative for growth after 5 days of incubation. This does not rule out bloodstream infection: automated blood culture systems often fail to detect growth of *M. hominis*. This failure is not completely understood, but might be attributed to the following: (a) BacT/ALERT (bioMérieux) and BACTEC (BD) blood cultures contain anticoagulant sodium polyanethol sulfonate, which may inhibit *Mycoplasma*, and therefore, the growth may not exceed the threshold value for positivity, resulting in the negative blood culture [13, 14], or (b) despite growth in the blood culture, the unique metabolic properties of *M. hominis* create very low amounts of CO_2_ which are insufficient to trigger the automated blood culture system [13]. As such, molecular methods are most reliable for *M. hominis* detection. Several in-house real-time PCR systems [15, 16], and some commercial systems [17], have been developed, showing increased sensitivity and specificity of *M. hominis* detection in clinical specimens in comparison to culture.

The growth of *M. hominis*, usually found in urogenital samples, in two different intraoperative samples within a very brief time frame of only 2 weeks was very unusual. For this reason, we initiated further investigation including WGS-typing of both *M. hominis* isolates. WGS provides the high resolution for epidemiological investigations [18] and has previously been used in an epidemiological investigation of *M. hominis* [19]. In our case report, the results of the cgMLST analysis shows that isolates from patient 1 and patient 2 were identical, highly suggestive of a common source (Fig. 3). Therefore, the infection is likely to have originated from the organ donor. Unfortunately, no clinical data on the colonization/infection status of the donor were available. The main risk factor for infection in both patients was probably the immunosuppression. A liver transplantation from the same deceased donor was performed without any postoperative complications, and we therefore hypothesize that the donor was colonized/infected urogenitally. The possibility of *Mycoplasma* infection in allograft recipients should be raised among the clinicians, especially if cultures appear negative, and there is no improvement under broad-spectrum beta-lactam antimicrobial therapy.

Serological screening for certain infectious agents in deceased organ and living kidney donors is mandatory. Depending on clinical circumstances, additional microbiological cultures are sometimes required, but they do not routinely include screening for urogenital *Mollicutes*. The prevalence of *M. hominis* in sexually active males can be as high as 20%, and 3.1–15% in females, depending on methodological and true population differences [1]. Although cases of non-sexual transmission have been described [20], most cases of genital colonization with *Mollicutes* among adults occur as a result of sexual contact and are correlated with the number of sexual partners [21, 22]. For this reason, screening of young, sexually active deceased and living kidney donors could help prevent immunosuppression-associated post-transplant infections with these facultative pathogens. Gerber and colleagues suggest screening of renal allograft recipients for urogenital *Mycoplasma* spp. and *Ureaplasma* spp. in symptomatic urinary tract infections including pyelonephritis with negative standard urine cultures, signs or symptoms of urethritis, graft dysfunction in a patient with concomitant sterile leukocyturia, or deep wound infections with negative or unconvincing standard cultures [4]. Neither of our patients was screened urogenitally for presence of *M. hominis*.

In conclusion, transplant patients with no improvement of febrile infection under broad-spectrum beta-lactam antibiotics with negative bacterial cultures should raise suspicion for infection with *Mycoplasma* or other *Mollicutes*. In such cases, a specific PCR from the intraoperative samples should be requested by the clinician. A general screening of the young, sexually active donors for genital *Mollicutes* is an issue that deserves further discussion, in particular if kidney transplantation is considered. WGS proved to be an excellent tool for epidemiologic investigations even for fastidious and slow-growing bacterial pathogens such as *Mollicutes*.

## Supplementary Information

ESM 1 (XLSX 22 kb)

## Data Availability

Not applicable.
